# Preferential occupancy of Eu^3+^ and energy transfer in Eu^3+^ doped Sr_2_V_2_O_7_, Sr_9_Gd(VO_4_)_7_ and Sr_2_V_2_O_7_/Sr_9_Gd(VO_4_)_7_ phosphors†

**DOI:** 10.1039/c7ra08089a

**Published:** 2018-01-03

**Authors:** Ling Li, Wenjun Wang, Yu Pan, Yuhan Zhu, Xiaoguang Liu, Hyeon Mi Noh, Byung Kee Moon, Byung Chun Choi, Jung Hyun Jeong

**Affiliations:** Hubei Collaborative Innovation Center for Advanced Organochemical Materials, Ministry of Education Key Laboratory for the Synthesis and Applications of Organic Functional Molecules, Hubei University Wuhan 430062 China; Department of Physics, Pukyong National University Busan 608-737 Korea

## Abstract

The vanadate-based phosphors Sr_2_V_2_O_7_:Eu^3+^ (SV:Eu^3+^), Sr_9_Gd(VO_4_)_7_:Eu^3+^ (SGV:Eu^3+^) and Sr_9_Gd(VO_4_)_7_/Sr_2_V_2_O_7_:Eu^3+^ (SGV/SV:Eu^3+^) were obtained by solid-state reaction. The bond-energy method was used to investigate the site occupancy preference of Eu^3+^ based on the bond valence model. By comparing the change of bond energy when the Eu^3+^ ions are incorporated into the different Sr, V or Gd sites, we observed that Eu^3+^ doped in SV, SGV or SV/SGV would preferentially occupy the smaller energy variation sites, *i.e.*, Sr4, Gd and Gd sites, respectively. The crystal structures of SGV and SV, the photoluminescence properties of SGV:Eu^3+^, SV, SGV/SV and SGV/SV:Eu, as well as their possible energy transfer mechanisms are proposed. Interesting tunable colours (including warm-white emission) of SGV/SV:Eu^3+^ can be obtained through changing the concentration of Eu^3+^ or changing the relative quantities of SGV to SV by increasing the calcination temperature. Its excitation bands consist of two types of O^2−^ → V^5+^ charge transfer (CT) bands with the peaks at about 325 and 350 nm respectively, as well as f–f transitions of Eu^3+^. The obtained warm-white emission consists of a broad photoluminescence band centred at about 530 nm, which originates from the O^2−^ → V^5+^ CT of SV, and a sharp characteristic spectrum (^5^D_0_–^7^F_2_) at about 615 and 621 nm.

## Abstract 2

The vanadate-based phosphors Sr_2_V_2_O_7_:Eu^3+^ (SV:Eu^3+^), Sr_9_Gd(VO_4_)_7_:Eu^3+^ (SGV:Eu^3+^) and Sr_9_Gd(VO_4_)_7_/Sr_2_V_2_O_7_:Eu^3+^ (SGV/SV:Eu^3+^) were obtained by solid-state reaction.

## Graphical abstract

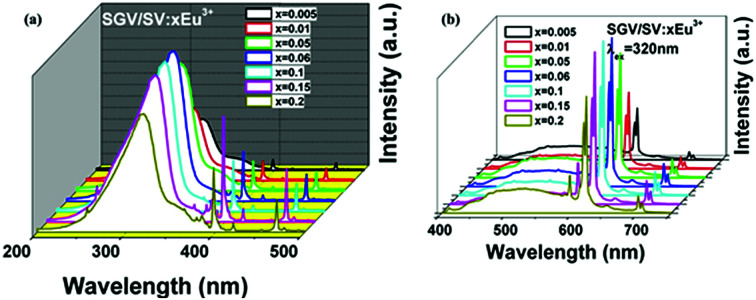

## 1. Introduction

Recently, vanadate-based phosphors have drawn increasing attention due to the self-activated emitting properties of [VO_4_]^3−^ group, the sensitization from [VO_4_]^3−^ to rare earth ions as well as their long wavelength excitation and the excellent chemical stabilities.^1–8^ The vanadate group, namely, [VO_4_]^3−^, in which the central metal ion V is coordinated by four oxygen ligands in a tetragonal symmetry, exhibits broad and intense charge transfer (CT) absorption bands in the UV region and some of them can produce intense broadband CT emission spectra from 400 to more than 700 nm related to the local structure.^9–11^ When excited by UV light, these vanadates or rare earth ions-doped materials have the capability to convert the ultraviolet emission into white light.^4,12,13^

In general, the first essential factor that determines the luminescence quantum efficiency of vanadate-based phosphors originating from O^2−^ → V^5+^ CT transition is the distortion of the VO_4_ tetrahedron. The excitation process of O^2−^ → V^5+^ CT is always allowed; thus, most of the vanadates show self-activated properties, while the intersystem crossing (^1^T_1_, ^1^T_2_ → ^3^T_1_, ^3^T_2_) and luminescence process (^3^T_1_, ^3^T_2_ → ^1^A_1_) are forbidden in the ideal *T*_d_ symmetry due to the spin selection rule.^1^ For example, in the crystal YVO_4_, O^2−^ → V^5+^ CT luminescence process is forbidden and thus, the luminescence of O–V CT cannot be observed at room temperature because in this crystal, V atom is coordinated with four equal oxygens and shows ideal *T*_d_ symmetry (all four Y–O bond lengths are 1.7 Å).^14^ However, in Eu^3+^-doped YVO_4_, the O^2−^ → V^5+^ CT energy can effectively be transferred to Eu^3+^ and shows intense red photoluminescence corresponding to the electric dipole transition, ^5^D_0_ → ^7^F_2_, of Eu^3+^ ions.^15^ However, the structure of the VO_4_ tetrahedron has, to some extent, a distorted *T*_d_ symmetry as compared to that of an ideal tetrahedron; thus, these forbidden processes are partially allowed due to the spin–orbit interaction.^1,10^ The vanadates with this type of structure can show intense O^2−^ → V^5+^ CT emission. For example, AVO_3_ (A: Rb and Cs) exhibits intense broadband emission from 400 nm to more than 700 nm under UV excitation.^5,9,16^ M_2_V_2_O_7_ (M = Ca, Sr, Ba) with distorted *T*_d_ symmetry around V atoms can emit strong O^2−^ → V^5+^ CT luminescence.^17^ Although only a few Eu^3+^ doped with this type of vanadates, such as Ba_3_LiMgV_3_O_12_:Eu^3+^,^18^ showed white light, while most rare earth ions-doped with this type of vanadates only produced red, yellow or green or blue-green light; other examples include M_2_V_2_O_7_ (M = Ca, Sr, Ba):Eu^3+^,^19^ Li_2_Ca_2_ScV_3_O_12_:Eu^3+^,^6^ and Ba_2_Y_2/3_V_2_O_8_:Eu^3+^.^13^

The vanadate phosphors exhibit two types of important advantages according to the symmetrical characteristic of VO_4_: first, self-activated emission arising from O^2−^ → V^5+^ CT with distorted *T*_d_ symmetry and second, the efficient energy transfer from self-activated O^2−^ → V^5+^ CT to Eu^3+^ with *T*_d_ symmetry.^1^ It is very interesting and important to investigate the preferential occupancy of Eu^3+^ and PL properties in the mixed phosphor. This is because the Sr_9_Gd(VO_4_)_7_ and Sr_2_V_2_O_7_ vanadates show two different types of important advantages according to the symmetrical characteristic of VO_4_. Sr_2_V_2_O_7_ shows self-activated emission arising from O^2−^ → V^5+^ CT with distorted *T*_d_ symmetry, but the energy transfer from O^2−^ → V^5+^ CT to the Eu ions is not effective. However, Eu^3+^ doped Sr_9_Gd(VO_4_)_7_ shows efficient energy transfer from self-activated O^2−^ → V^5+^ CT to Eu^3+^, but Sr_9_Gd(VO_4_)_7_ could not produce self-activated emission due to the symmetrical characteristic of VO_4_ with *T*_d_ symmetry. Eu^3+^ doped Sr_2_V_2_O_7_/Sr_2_Gd(VO_4_)_7_ possesses the two advantages of self-activated emission and efficient energy transfer from the host lattice to Eu^3+^. In order to investigate the structure and the photoluminescence of vanadate phosphors, we synthesized Sr_2_V_2_O_7_ (SV), Sr_9_Gd(VO_4_)_7_:Eu^3+^ (SGV:Eu^3+^) and Sr_9_Gd(VO_4_)_7_/Sr_2_V_2_O_7_:Eu^3+^ (SGV/SV:Eu^3+^); in particular, Sr_9_Gd(VO_4_)_7_/Sr_2_V_2_O_7_:Eu^3+^ can possess both the above advantages of vanadates. The occupying sites of Eu^3+^, photoluminescence properties and the relationship between O^2−^ → V^5+^ CT energy and crystal structure are discussed. The photoluminescence and excitation mechanism as well as energy transfer phenomenon between the host lattice and Eu^3+^ are investigated.

## 2. Experimental section

### 2.1. Materials and synthesis

The phosphor with nominal composition Sr_9_Gd(VO_4_)_7_:5% Eu^3+^ was synthesized using a high-temperature solid-state reaction method from a stoichiometric mixture of SrCO_3_ (99.9%), V_2_O_5_ (99.9%), Gd_2_O_3_ (99.99%), and Eu_2_O_3_ (99.99%). The mixture was ground in alumina crucibles, homogeneously mixed; finally the mixtures were heated at 1023 K for 12 h and at 1223 K for 12 h and then cooled down to room temperature to obtain a white powder. Sr_2_V_2_O_7_/Sr_9_Gd(VO_4_)_7_:5% Eu^3+^ can be obtained when heated at 1123 K for 12 h.

Pure Sr_2_V_2_O_7_:Eu^3+^ was prepared using traditional solid-state method from a stoichiometric mixture of SrCO_3_ (99.9%), V_2_O_5_ (99.9%) and Eu_2_O_3_ (99.99%). After grinding in an alumina crucible and mixing homogeneously, the mixture was heated at 1323 K for 24 h and then cooled down to room temperature to obtain a white powder.

### 2.2. Characterizations

Powder X-ray diffraction (XRD) measurements were recorded on a D/MAX 2500 instrument (Rigaku) with a Rint 2000 wide angle goniometer and Cu Kα1 radiation (*λ* = 1.54056 Å) at 40 kV and 100 mA. The diffraction patterns were scanned over an angular (2*θ*) range of 20−80° at intervals of 0.02° with a counting time of 0.6 s per step. Photoluminescence (PL) studies were conducted on a fluorescence spectrophotometer (Photon Technology International) equipped with a 60 W Xe-arc lamp as the excitation light source. All the measurements were recorded at room temperature.

### 2.3. Theoretical method

Based on the chemical bond viewpoint, the dopants preferentially occupy the sites with smaller alterations of bond energy.^20^ The variation of bond energy can be measured by the following expression1
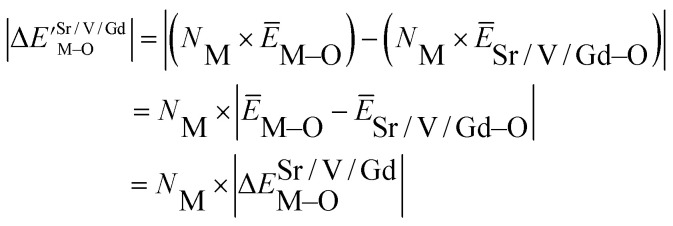
where *N*_M_ is the dopant content in the unit of mol. For the same host lattice, the *N*_M_ is the same, so when we discuss the occupancy of any anion, we only analyse the Δ*E*^Sr/V/Gd^_M–O_ value do not consider the 
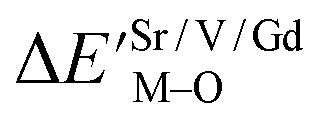
 value. *Ē*_M–O_ and *Ē*_Sr/V/Gd–O_ are the mean bond energies of M–O bond and Sr–O or V–O or Gd–O, which can be expressed as2
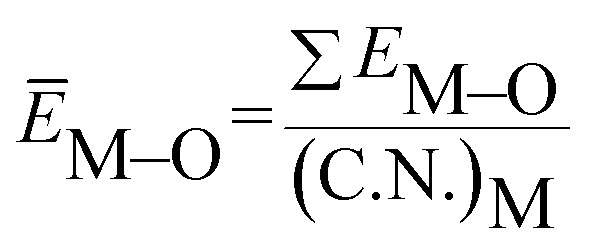
3
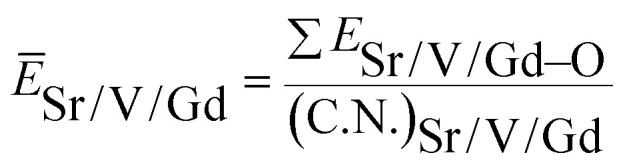


The total bond energy, ∑*E*_Sr/V/Gd–O_, in kcal of the crystals can be regarded as a bond-energy sum of all constituent chemical bonds. For example, in Sr_2_V_2_O_7_, there are four different sites,^21^ that is, Sr1, Sr2, Sr3 and Sr4. Taking Sr1 site as an example, there are eight types of constituent Sr1–O, that is, Sr1–O1, Sr1–O2, Sr1–O6 (there are two Sr1–O6 bonds with different distance), Sr1–O8, Sr1–O9, Sr1–O11 and Sr1–O14. Thus, the bond energy, ∑*E*_Sr1–O_, can be calculated using the following formula:4
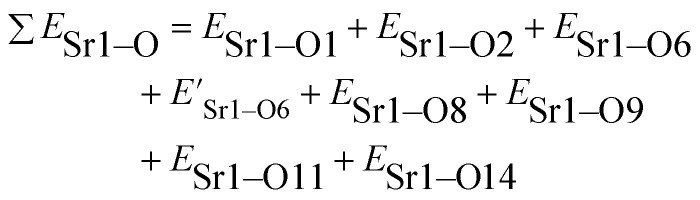


The ∑*E*_M–O_ is the sum of bond energies of different dopants in the crystallographic frame. When the dopant ion is Eu^3+^, *E*_M–O_ can be estimated through the following equation^20^5
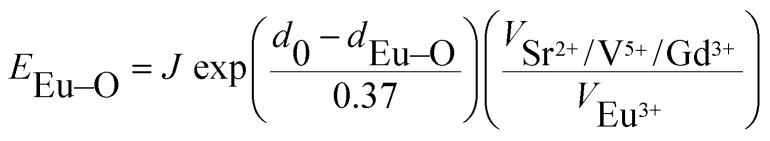
where *V*_Sr^2+^/V^5+^/Gd^3+^_ presents the valence state of Sr or V or Gd and *V*_Eu^3+^_ is the dopant valence of Eu^3+^. This indicates that the valence state has influence on the crystal bond energy if the valence state of the dopant is not equal to that of the original ions. The coefficient *J* is equal to the standard atomization energy, which can be estimated using the following formula:^22^6
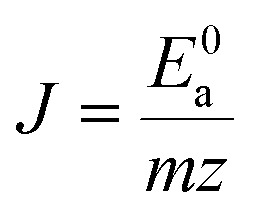
where *m* is the number of cations in the formal molecule, *z* is the cation valence and *E*^0^_a_ is molar atomization energy (kcal mol^−1^) of an oxide crystal M_*m*_O_*n*_ at the standard state (normal pressure, 298 K). *E*^0^_a_ may be expressed as7
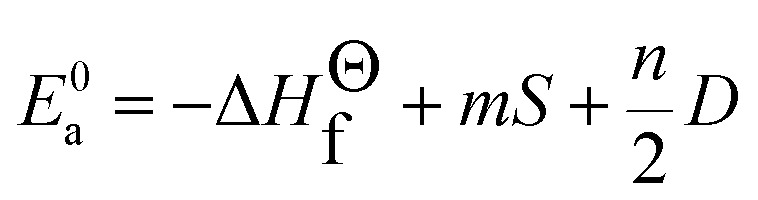
where Δ*H*^*Θ*^_f_ is the standard heat of formation of M_*m*_O_*n*_ (−592, −1550, −1816 and −1652 kJ mol^−1^ for SrO, V_2_O_5_, Gd_2_O_3_ and Eu_2_O_3_, respectively), *S* is the heat of metal sublimation or, more generally, heat of atomization of M (164, 515, 397.5 and 177.4 kJ mol^−1^ for Sr, V, Gd and Eu, respectively) and *D* is the heat of dissociation of O_2_ molecule (493.804 kJ mol^−1^). According to the equation above, we can determine that the *J* values of Sr^2+^–O, V^5+^–O, Gd^3+^–O and Eu^3+^–O are 119.80, 91.13, 133.45 and 109.40 kcal mol^−1^, respectively.

In eqn (5), *d*_0_ is an empirically determined parameter, which is measured by processing all available crystallographic data; it is constant for a given atom pair.^23^ The *d*_0_ of Sr^2+^–O^2−^, V^5+^–O^2−^ and Eu^3+^–O^2−^ are 2.118, 1.917, and 2.074 Å, respectively. Furthermore, the *d*_0_ value of Gd^3+^–O^2−^ can be estimated by the formula below:

8 *d*_0_ = *r*_c_ + *A* × *r*_a_ + *P* − *D* − *F*

where *r*_c_ and *r*_a_ are contributions to *d*_0_ from the cation and anion, respectively. The multiplier *A* is set to 0.8 for transition metal ions with d electrons or else it is set to 1.0. *P*, *D* and *F* are corrections required when cation contains non-bonding p, d and f electrons, respectively. The *r*_c_, *r*_a_ and *D* values can be obtained in the ref. 23. *P* and *F* can be calculated using9
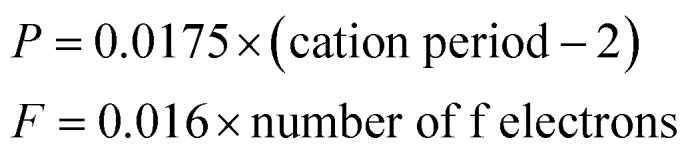


For lanthanide ions, the calculated *d*_0_ values using (8) are larger 0.079 than those obtained in the ref. 23. Hence, we corrected the formula (8) to be as below:10
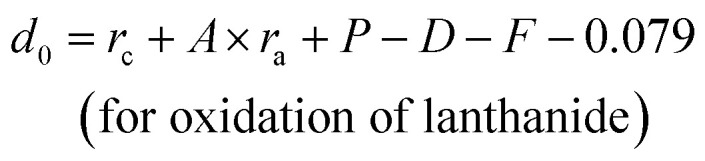
Hence, the *d*_0_ of Gd^3+^–O^2−^ is 2.049 Å.

## 3. Results and discussion

### 3.1. Phase identification of Sr_2_V_2_O_7_:Eu^3+^, Sr_9_Gd(VO_4_)_7_/Sr_2_V_2_O_7_:Eu^3+^ and Sr_9_Gd(VO_4_)_7_:Eu^3+^

**Fig. 1:**
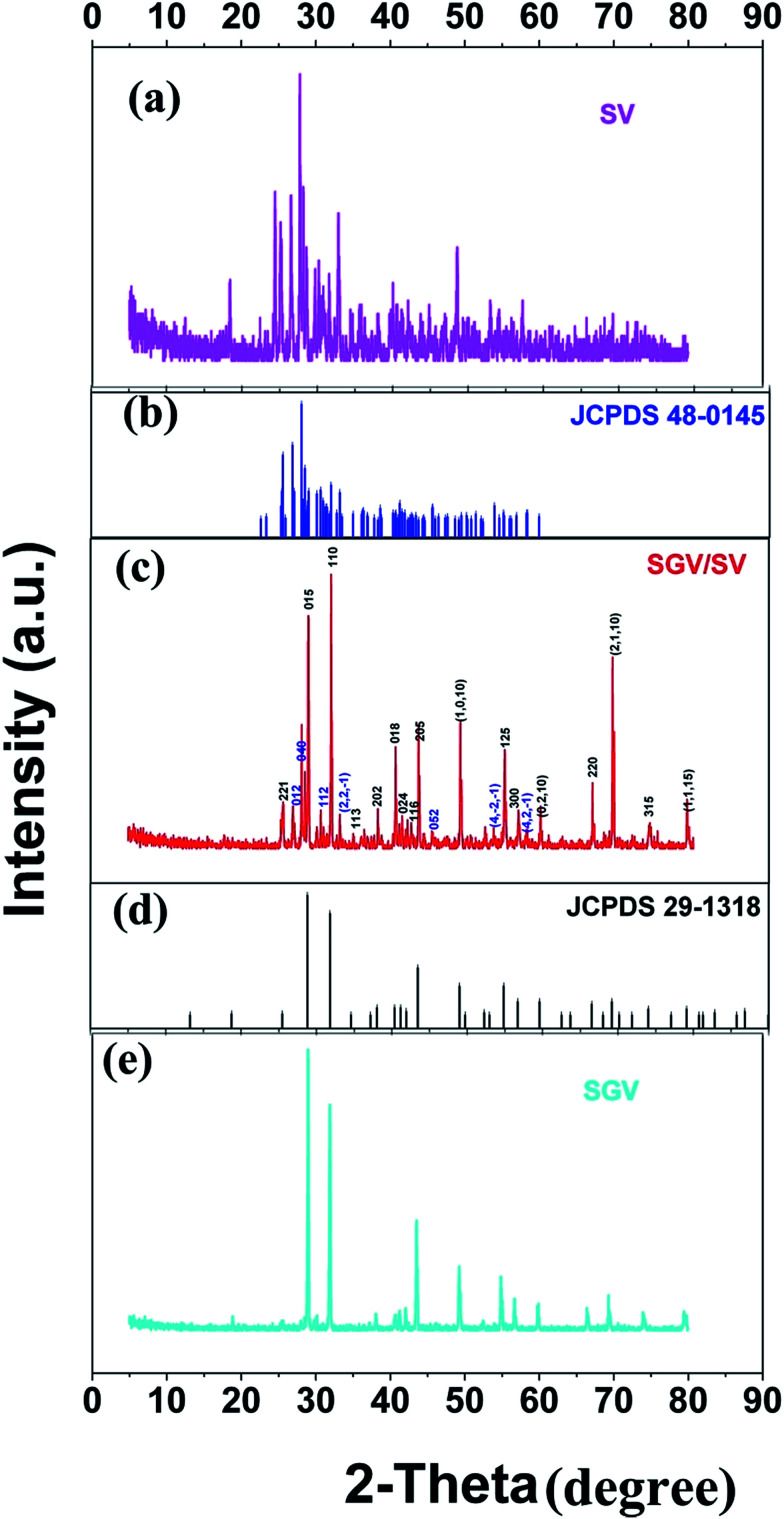
(a) XRD patterns of Sr_2_V_2_O_7_, and (b) the JCPDS card 48-0145 of Sr_2_V_2_O_7_ (blue color), (c) XRD patterns of Sr_9_Gd(VO_4_)_7_/Sr_2_V_2_O_7_:5% Eu^3+^ (red color); (d) the JCPDS card 29-1318 of Sr_3_(VO_4_)_2_ (pure Sr_9_Gd(VO_4_)_7_:5% Eu^3+^ phase is isotypic with Sr_3_(VO_4_)_2_) (black color) as well as (e) XRD patterns of Sr_9_Gd(VO_4_)_7_.

Fig. 1 shows the representative X-ray diffraction patterns for Sr_2_V_2_O_7_:Eu, Sr_9_Gd(VO_4_)_7_/Sr_2_V_2_O_7_:Eu and Sr_9_Gd(VO_4_)_7_:Eu samples. Fig. 1(a) shows the XRD patterns for Sr_2_V_2_O_7_:Eu. All the diffraction peaks of Sr_2_V_2_O_7_:Eu can be indexed to the reported JCPDS 48-0148 card (60° > 2*θ* > 20°). It can be demonstrated that the obtained Sr_2_V_2_O_7_:Eu sample is pure. Fig. 1(e) shows that the XRD patterns of Sr_9_Gd(VO_4_)_7_:Eu are similar to those of Sr_3_(VO_4_)_2_ (Fig. 1(d), JCPDS file number 29-1318);^24^ no peaks from other phases such as Gd_2_O_3_, SrO, or V_2_O_5_ appear. The structure of Sr_9_Gd(VO_4_)_7_:Eu is similar to that of Sr_9_Lu(VO_4_)_7_, which is found to be isotypic with Ca_3_(VO_4_)_2_ (ref. 25) or Sr_3_(VO_4_)_2_.^24^ The above results indicate the formation of a pure Sr_9_Gd(VO_4_)_7_:Eu. The XRD patterns of Sr_9_Gd(VO_4_)_7_/Sr_2_V_2_O_7_:Eu component is clearly shown in Fig. 1(c). The detailed crystal plane diffraction peaks ascribed to Sr_2_V_2_O_7_ and Sr_9_Gd(VO_4_)_7_:Eu are labeled and distinguished using blue and red colors, respectively. Except the diffraction peaks arising from Sr_2_V_2_O_7_ and Sr_9_Gd(VO_4_)_7_ crystals, no other peaks can be found, which indicates that we have obtained the component of “pure” Sr_9_Gd(VO_4_)_7_/Sr_2_V_2_O_7_. From Fig. 1(c) and (e), we can judge that the mechanism of producing Sr_2_V_2_O_7_, Sr_9_Gd(VO_4_)_7_/Sr_2_V_2_O_7_, and Sr_9_Gd(VO_4_)_7_ is as below:

11 2SrCO_3_ + V_2_O_5_ ⇔ Sr_2_V_2_O_7_ + 2CO_2_↑

12 18SrCO_3_ + Gd_2_O_3_ + 7V_2_O_5_ ⇔ 3.5*x*Sr_2_V_2_O_7_ + 0.5*x*Gd_2_O_3_ + 2*x*SrO + (2 − *x*)Sr_9_Gd(VO_4_)_7_ + 18CO_2_ ⇔ 2Sr_9_Gd(VO_4_)_7_ + 18CO_2_↑

From mechanism (12), it can be shown that there are some Gd_2_O_3_ and SrO except for the main products of Sr_9_Gd(VO_4_)_7_/Sr_2_V_2_O_7_; however, it cannot be observed in Fig. 1(c) because the relative quantities of Gd_2_O_3_ and SrO are so small that XRD patterns cannot be indexed. In our investigation, we ignored the existence of the samples Gd_2_O_3_ and SrO because they do not influence the photoluminescence of Sr_9_Gd(VO_4_)_7_/Sr_2_V_2_O_7_:Eu.

### 3.2. Photoluminescence properties

#### 3.2.1. Site occupancy and PL properties of Sr_2_V_2_O_7_:Eu

**Fig. 2:**
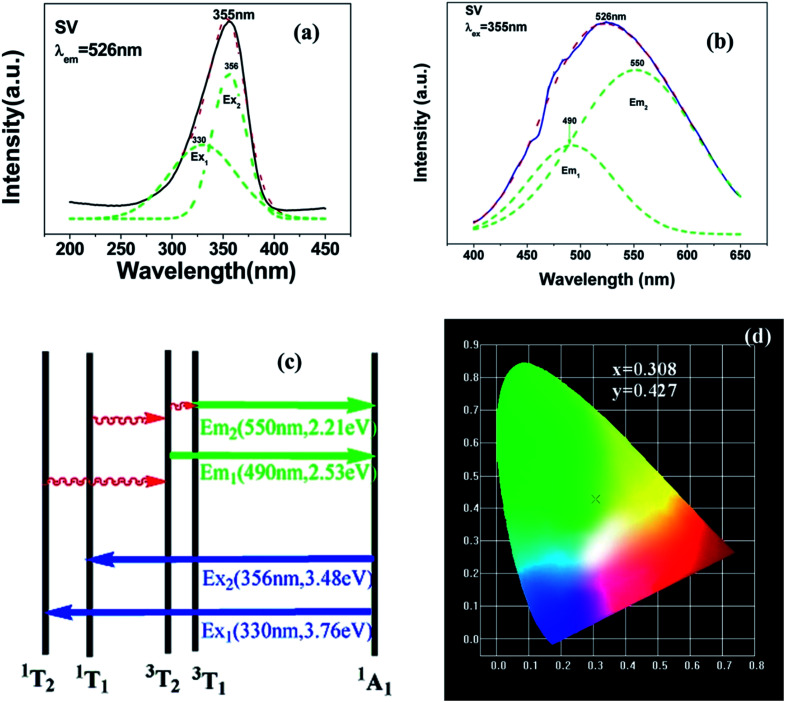
PL excitation spectra (a) and emission spectra (b) of Sr_2_V_2_O_7_. Red dotted lines indicate excitation spectra or emission spectra fitted with two Gaussian curves (green dotted lines) corresponding to excitation bands Ex_1_, Ex_2_ and emission bands Em_1_, Em_2_, respectively. (c) Schematic model of absorption and emission processes of [VO_4_]^3−^ tetrahedron in Sr_2_V_2_O_7_. Ex_1_ and Ex_2_ represent excitation processes ^1^A_1_ → ^1^T_1_ and ^1^A_1_ → ^1^T_2_, respectively. Em_1_ and Em_2_ represent emission processes ^3^T_2_ → ^1^A_1_ and ^3^T_1_ → ^1^A_1_, respectively. (d) CIE chromaticity diagram shows colour coordinates of the luminescence of Sr_2_V_2_O_7_.

The PL properties of pure Sr_2_V_2_O_7_ were reinvestigated for the occupancy of Eu^3+^ in SV sample. Fig. 2 shows the PL excitation and emission spectra of pure Sr_2_V_2_O_7_ at room temperature. Upon monitoring the wavelength at 526 nm, Sr_2_V_2_O_7_ shows a broad excitation band with peak at 355 nm spanning from 200 to 400 nm, which can be matched well with the excitation band of UV-LED chips. The excitation spectrum originates from O^2−^ → V^5+^ CT transition, which can be deconvoluted into two peaks at 330 (3.76 eV) and 356 nm (3.48 eV) corresponding to the ^1^A_1_ → ^1^T_2_ and ^1^A_1_ → ^1^T_1_ transition of VO_4_^3−^ group, respectively, as shown in Fig. 2(a) and (c). The values are listed in Table 2. In Fig. 2(a), red dotted lines indicate excitation spectra fitted with two Gaussian curves (green dotted lines) corresponding to two excitation bands: Ex_1_ and Ex_2_. The energy difference between the peaks Ex_1_ and Ex_2_ is 0.28 eV, which is ascribed to the energy difference between ^1^T_2_ and ^1^T_1_ as reported in many ref. 6, 18 and 26. The corresponding excitation and emission mechanisms are shown in Fig. 2(c). Under the excitation of 355 nm UV-light irradiation, Sr_2_V_2_O_7_ shows a strong green emission (Fig. 2(b)) due to the typical V–O charge transfer (CT) emission of VO_4_^3−^ ions, consisting of a strong broad band (400–650 nm) with a maximum at 526 nm. Fig. 2(d) shows a graphic of the Commission Internationale de L'Eclairage (CIE) 1931 chromaticity coordinate of pure Sr_2_V_2_O_7_ phosphors excited at 325 nm. The (*x*, *y*) chromaticity coordinate of Sr_2_V_2_O_7_ is (0.308, 0.427) in the green region. The emission spectrum can be further fitted by two Gaussian components with two peaks at Em_1_ (490 nm, *i.e.* 2.53 eV) and Em_2_ (550 nm, *i.e.* 2.25 eV), which are labeled using green color of Fig. 2(b). The energy gap between the two peaks is 0.28 eV, which is ascribed to the energy difference between ^3^T_1_ and ^3^T_2_ as reported in many ref. 6, 18 and 26.

**Fig. 3:**
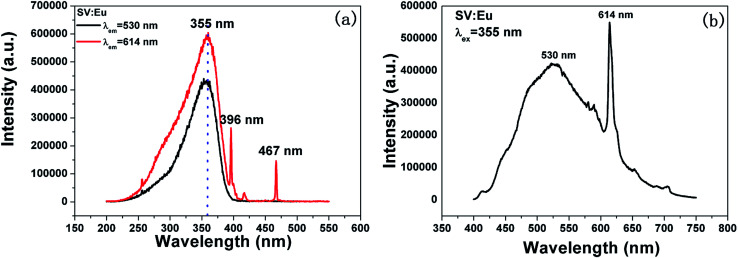
PL excitation spectra (a) and emission spectra (b) of Sr_2_V_2_O_7_:5% Eu (SV:Eu).

Fig. 3 shows the PL properties of Eu^3+^ in SV, which are different in the ref. 2 and 19. The emission spectrum (excited at 355 nm) is shown in Fig. 3(b). The emission spectrum contains a broadband emission in the 400–590 nm wavelength region with a maximum at about 530 nm and a sharp peak at 614 nm. The broad peak has a 30 nm redshift compared with the reported value of 500 nm, which is very large, but the breadth and peak of the broad band emission is similar to that of SV. This corresponds to the charge transfer from the O^2−^ to V^5+^ localized within the tetrahedrally coordinated VO_4_^3−^ group. The sharp peak at 614 nm originates from the ^5^D_0_ → ^7^F_2_ transition in Eu^3+^ dopant.

In Fig. 3(a), the excitation spectrum monitored at 614 nm has a broadband in the 250–400 nm wavelength region with the peak at 355 nm, which is close to the excitation spectrum monitored at 530 nm. This indicates that the broad excitation band arises due to the charge transfer from oxygen 2p orbital of O^2−^ to an empty d orbital of V^5+^, and not to the orbital of Eu^3+^.

Coordination atoms and their bond lengths of four sites of Sr and four sites of V are listed in columns 2 and 3 of Table 1, respectively. All calculated 
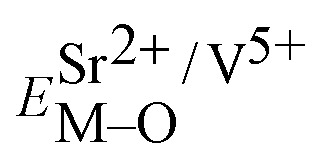
, 
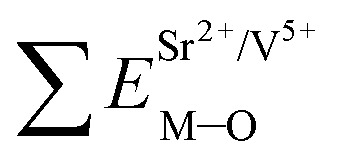
 and Δ*E*^Sr/V^_M–O_ values of various possible substituted ions including Sr^2+^ and V^5+^ on both Sr^2+^ and V^5+^ sites are listed in Table 1. These results indicate that the value of bond energy variation is in the order Sr4 < Sr2 < Sr3 < Sr1 < V1 < V2 < V3 < V4. According to the bond energy method, Eu^3+^ ions should preferentially occupy the sites with smaller alterations of bond energy values, Δ*E*^Sr/V^_Eu–O_. Within this energy formation argument, the priority site for the Eu^3+^ incorporation of the luminescent centers is Sr4.

**Table 1:** Single and sum bond energies of Sr–O or V–O bonds in Sr_2_V_2_O_7_ (
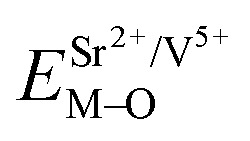
and 
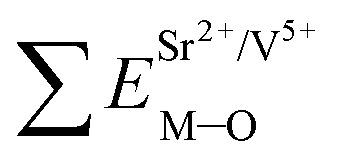
), single and sum bond energies of Eu–O bonds in Sr_2_V_2_O_7_:Eu (
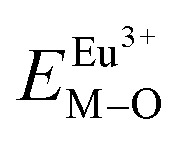
 and 
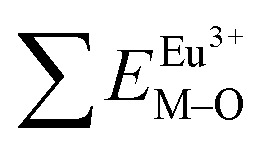
) and variation of bond energy (Δ*E*^Sr/V^_M–O_) when the Eu^3+^ ion is located at different Sr^2+^ and V^5+^ sites. All units of bond energy are kcal mol^−1^

Central atom	Coordination atom	*d* (Å)	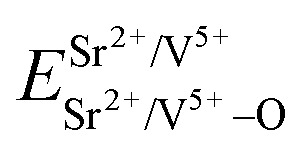	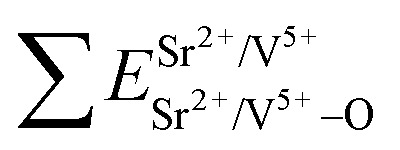	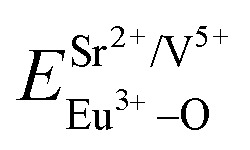	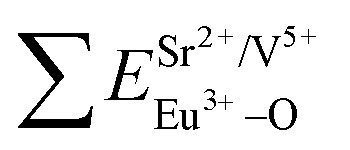	Δ*E*^Sr/V^_Eu–O_
Sr1	O14	2.4848	44.45	254.1	24.03	137.3	15.84
	O1	2.5205	40.37		21.82		
	O6	2.5283	39.52		21.36		
	O6	2.5613	36.15		19.54		
	O11	2.5807	34.30		18.54		
	O2	2.7065	24.42		13.20		
	O9	2.7374	22.46		12.14		
	O8	2.9578	12.38		6.692		
Sr2	O10	2.5407	38.22	229.8	20.66	124.2	11.7
	O4	2.5415	38.14		20.62		
	O5	2.6112	31.59		17.08		
	O2	2.6231	30.59		16.54		
	O12	2.6275	30.23		16.34		
	O9	2.6771	26.44		14.29		
	O13	2.8247	17.74		9.589		
	O11	2.9948	11.20		6.055		
	O7	3.2494	5.629		3.043		
Sr3	O4	2.5307	39.27	234.9	21.23	127.0	12.0
	O2	2.6431	28.98		15.67		
	O10	2.6439	28.92		15.63		
	O1	2.6481	28.59		15.45		
	O7	2.6617	27.56		14.90		
	O13	2.6949	25.19		13.62		
	O10	2.7036	24.61		13.30		
	O5	2.8155	18.19		9.831		
	O8	2.9238	13.57		7.336		
Sr4	O5	2.5484	37.43	238.5	20.23	128.9	10.96
	O9	2.5537	36.90		19.95		
	O6	2.5653	35.76		19.33		
	O14	2.6598	27.70		14.97		
	O3	2.662	27.54		14.89		
	O8	2.7251	23.22		12.55		
	O13	2.7647	20.86		11.28		
	O11	2.8376	17.13		9.261		
	O7	3.1886	6.635		3.586		
	O1	3.2685	5.346		2.890		
V1	O14	1.6626	133.2	465.4	554.3	1937	368
	O13	1.6828	126.1		524.9		
	O9	1.7047	118.9		494.7		
	O3	1.8192	87.23		363.0		
V2	O4	1.6677	131.4	467.1	546.7	1944	369
	O11	1.6693	130.8	—	544.4		
	O2	1.7114	116.7	—	485.8		
	O12	1.8151	88.20	—	367.1		
V3	O7	1.6572	135.1	468.1	562.5	1948	370
	O10	1.6868	124.8		519.2		
	O5	1.6959	121.7		506.6		
	O12	1.8222	86.52		360.1		
V4	O8	1.6597	134.2	474.2	558.7	1974	375
	O1	1.6758	128.5		534.9		
	O6	1.695	122.0		507.8		
	O3	1.81	89.42		372.2		

#### 3.2.2. Site occupancy and PL properties of SGV and SGV:Eu

**Fig. 4:**
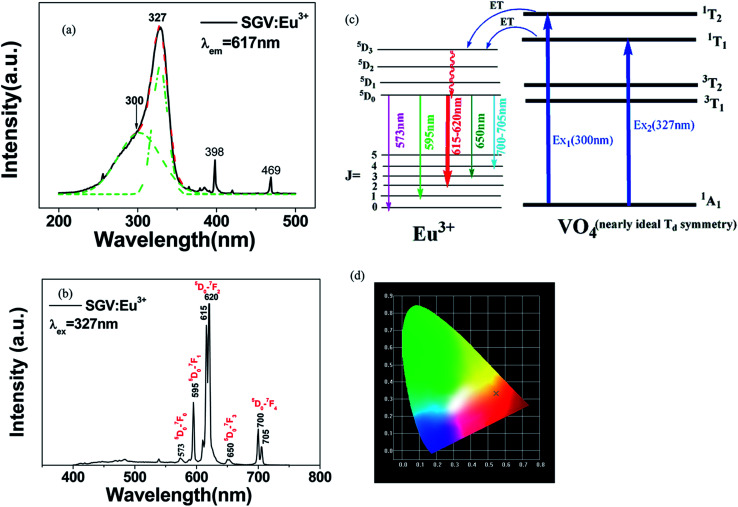
PL excitation spectra (a) and emission spectra (b) of SGV:5% Eu^3+^. Red dotted lines indicate excitation spectra fitted with two Gaussian curves (green dotted lines) corresponding to excitation bands Ex_1_, Ex_2_. (c) Schematic model of excitation, energy transfer and emission processes of VO_4_ tetrahedron in SGV:Eu^3+^. Ex_1_ and Ex_2_ represent excitation processes ^1^A_1_–^1^T_1_ and ^1^A_1_–^1^T_2_, respectively. (d) CIE chromaticity diagram showing color coordinates of the luminescence of SGV:Eu^3+^.

PL emission of pure SGV cannot be observed at room temperature, but Eu^3+^-doped SGV can emit strong red light. Fig. 4(a) and (b) show the typical excitation and emission spectra of SGV:Eu. The excitation spectrum of SGV:Eu obtained by monitoring the ^5^D_0_ → ^7^F_2_ at 617 nm is shown in Fig. 4(a). It consists of a broad excitation band with peak at 327 nm spanning from 200 to 350 nm, which can be matched well with the UV-LED chips, along with some dominated sharp lines in the wavelength region of 350 to 500 nm, which arise due to the characteristic f–f transition of Eu^3+^ at about 398 and 469 nm. The excitation is the typical O^2−^ → V^5+^ CT transition spectrum of [VO_4_]^3−^, which can be separated into two peaks at 300 and 327 nm corresponding to the ^1^A_1_ → ^1^T_2_ and ^1^A_1_ → ^1^T_1_ transition of [VO_4_]^3−^ group, respectively. This also indicates that the energy transfer can be taken place efficiently from VO_4_^3−^ to Eu^3+^ ions in SGV:Eu. The mechanism of energy transfer and luminescence of Eu^3+^ are shown in Fig. 4(c), which is similar to the energy transfer from [VO_4_]^3−^ to Eu^3+^ ions in YVO_4_:Eu.^15,27^

Comparing the O–V CT energy in pure sample SV (355 nm), the CT energy of [VO_4_]^3−^ in pure SGV (327 nm) is much higher. This is because the crystal field or environmental factor (*h*_e_) around V atoms in the two samples is different.^28^

Fig. 4(b) shows the emission spectra of SGV:Eu excited at 327 nm. The SGV:Eu phosphor shows bright red color. The (*x*, *y*) chromaticity coordinate of SGV is (0.547, 0.333) in the red region. Fig. 4(d) shows a graphic of the CIE 1931 chromaticity coordinate of pure SGV phosphors excited at 327 nm. As shown in Fig. 4(b), the dominant red emission bands of 615 and 620 nm are attributed to the electric dipole transition ^5^D_0_ → ^7^F_2_, indicating that Eu^3+^ ions are located at the sites of non-inversion symmetry. The emission peaks at about 573, 595, 650, and 700–705 nm are derived from the transition of ^5^D_0_ → ^7^F_0_, ^5^D_0_ → ^7^F_1_, ^5^D_0_ → ^7^F_3_, and ^5^D_0_ → ^7^F_4_, respectively, which are much weaker than the intensity of ^5^D_0_ → ^7^F_2_.

Consequently, ^5^D_0_ → ^7^F_2_ red emission (615 and 620 nm) presents the most prominent intensity in the emission spectrum. In SGV:Eu, the structure of SGV is isotypical with that of Sr_9_Lu(VO_4_)_7_, so Sr_9_Gd(VO_4_)_7_ can be analyzed according to the structure in the reference. In Sr_9_Gd(VO_4_)_7_, there are three different Sr or V sites and one Gd site; their coordination atoms are summarized in Table 3.

All calculated 
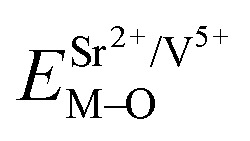
, 
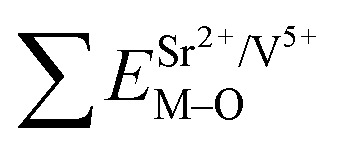
 and Δ*E*^Sr/V^_Eu–O_ values of various possible substituted ions including Sr^2+^, Gd^3+^and V^5+^ on both Sr^2+^, Gd^3+^and V^5+^ sites are listed in Table 3. The result indicates that the value of bond energy variation is in the order Gd1 < Sr3 < Sr1 < Sr2 < V2 < V3 < V1. According to the bond energy method, Eu^3+^ ions should preferentially occupy the sites with smaller alterations of bond energy values Δ*E*^Sr/Gd/V^_Eu–O_. Within this energy formation argument, the priority site for the Eu^3+^ incorporation of the luminescent centers is Gd.

#### 3.2.3. Site occupancy and PL properties of SGV/SV and SGV/SV:Eu^3+^

On the basis of the above PLE spectra of SGV:Eu^3+^ and SV, it can be observed that both of them have a broad absorption range in the UV region. Eu^3+^-doped SGV exhibits efficient energy transfer from [VO_4_]^3−^ to Eu^3+^ and emits strong red light and SV can emit strong green light. According to the calculated Δ*E*^Sr/Gd/V^_Eu–O_, in the “pure” SGV/SV system, the result indicates that the smallest value of bond energy variation would be at the Gd site. Consequently, we designed Eu^3+^-doped “pure” SGV/SV system expecting that Eu^3+^ can enter the site of SGV and emit strong red light, while SV can retain its strong green light and thus, the composition of red and green will produce white light or exhibit excellent luminescence properties.

**Fig. 5:**
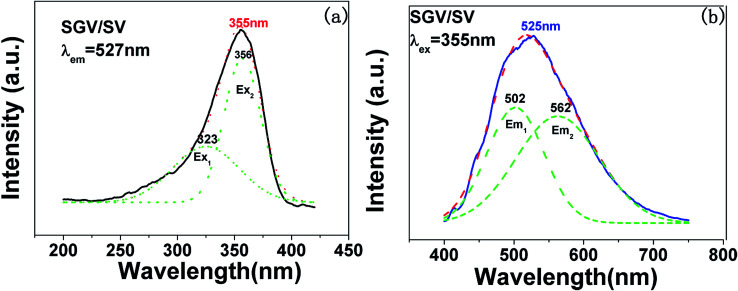
PL excitation spectra (a) and emission spectra (b) of Sr_9_Gd(VO_4_)_7_/Sr_2_V_2_O_7_. Red dotted lines indicate excitation spectra or emission spectra fitted with two Gaussian curves (green dotted lines) corresponding to excitation bands Ex_1_, Ex_2_ and emission bands Em_1_, Em_2_, respectively.

The excitation and emission spectra of “pure” SGV/SV are shown in Fig. 5. The peaks, full wave at half maximum (FWHM), Ex_1_ and Ex_2_ as well as Em_1_ and Em_2_ of excitation and emission spectra for “pure” SGV/SV are listed in Table 2.

**Table 2:** Comparing peaks and full wave at half maximum (FWHM) of the excitation and emission spectra SV, SGV and SGV:Eu with those of the mixed-compound SGV/SV

Compound	Excitation spectrum (nm)			Emission spectrum (nm)		
	Peak position		FWHM	Peak position		FWHM
SV	355	330, 356	50	526	550, 490	140
SGV	327	300, 327	50	No	No	No
SGV/SV	355	323, 356	52	525	562, 502	137

**Table 3:** Single and sum bond energies of Sr–O or V–O bonds in Sr_9_Gd(VO_4_)_7_ (
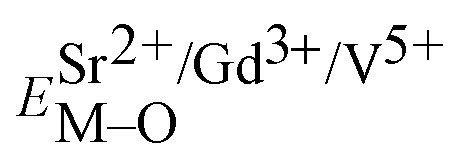
 and 
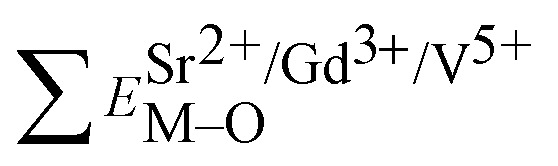
), single and sum bond energies of Eu–O bonds in Sr_9_Gd(VO_4_)_7_:Eu (
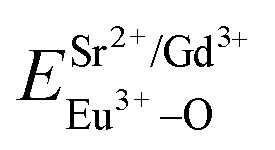
 and 
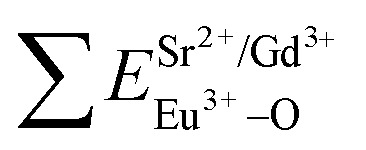
) and variation of bond energy (|Δ*E*|) when the Eu^3+^ ion is located at different Sr^2+^ and V^5+^ sites. All units of bond energy are kcal mol^−1^

Central atom	Coordination atom	*d* (Å)	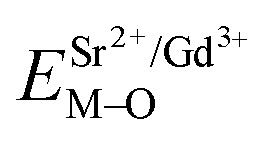	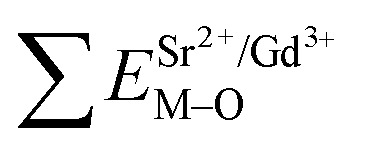	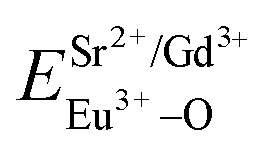	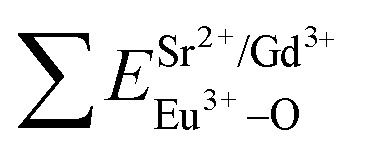	|Δ*E*|
Sr1	O8	2.4607	47.45	248.0	25.65	134.0	15.49
	O10	2.496	43.13		23.31		
	O7	2.5922	33.25		17.98		
	O2	2.6146	31.30		16.92		
	O5	2.6353	29.60		16.00		
	O6	2.6453	28.81		15.57		
	O6	2.7103	24.17		13.06		
	O4	3.0266	10.28		5.556		
Sr2	O5	2.5086	41.69	240.2	22.53	129.8	15.76
	O2	2.5139	41.09		22.21		
	O4	2.5867	33.75		18.24		
	O3	2.5871	33.72		18.22		
	O9	2.6318	29.88		16.15		
	O9	2.6856	25.84		13.97		
	O8	2.8354	17.23		9.316		
	O7	2.8411	16.97		9.173		
Sr3	O5	2.5899	33.46	216.5	18.09	117.0	11.05
	O4	2.5902	33.44		18.07		
	O7	2.6004	32.53		17.58		
	O1	2.7138	23.94		12.94		
	O10	2.7203	23.52		12.72		
	O10	2.7381	22.42		12.12		
	O8	2.7733	20.38		11.02		
	O3	2.8996	14.49		7.832		
	O2	2.9599	12.31		6.654		
Lu1/Gd1	O6	2.1762	72.32	401.3	83.00	460.5	9.89
	O6	2.1762	72.32		83.00		
	O6	2.1762	72.32		83.00		
	O9	2.2365	61.44		70.51		
	O9	2.2365	61.44		70.51		
	O9	2.2365	61.44		70.51		
V1	O1	1.6822	126.3	489.1	525.7	2036	386.8
	O2	1.6983	120.9		503.3		
	O2	1.6983	120.9		503.3		
	O2	1.6983	120.9		503.3		
V2	O3	1.6505	137.6	477.5	572.7	1988	377.5
	O5	1.7026	119.5		497.5		
	O4	1.7078	117.9		490.6		
	O6	1.7594	102.5		426.7		
V3	O7	1.6726	129.6	483.4	539.5	2012	382.3
	O10	1.687	124.7		518.9		
	O8	1.7157	115.4		480.2		
	O9	1.721	113.7		473.4		

In contrast with the values of pure SV, all the parameters are almost the same, which demonstrate that the emission with the peak at 525 nm comes from the SV in SGV/SV.

**Fig. 6:**
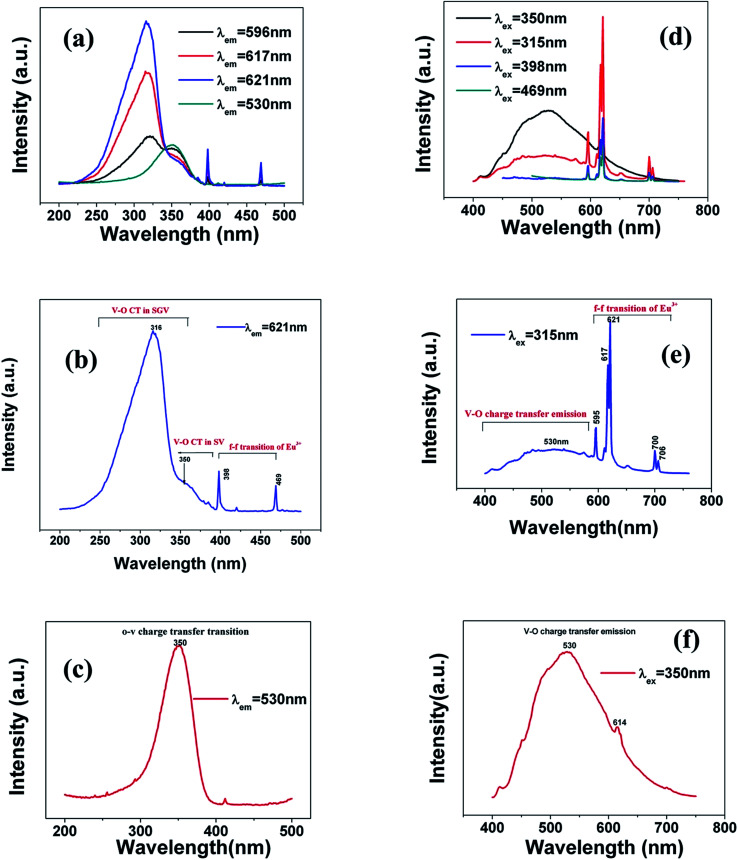
(a), (b) and (c) are excitation spectra of SGV/SV:Eu^3+^ and (d), (e) and (f) are emission spectra of SGV/SV:Eu^3+^ under different excitation spectra.

The typical excitation and emission of SGV/SV:5% Eu^3+^, represented by SGV/SV:5% Eu^3+^, monitored with different wavelengths and excited at different wavelengths are shown in Fig. 6(a) and (b). Similar excitation spectra with two strong broad bands (at about 320 and 350 nm) can be observed in SGV/SV:Ln^3+^ (Ln^3+^ = Sm^3+^, Dy^3+^, or Tm^3+^) as shown in Fig. S1.† It therefore can be demonstrated that the two broad excitation spectra of SGV/SV:Eu^3+^ arise from the O → V charge transfer of the host lattice, *i.e.*, SGV/SV and not from the CT transition of O^2−^ → Eu^3+^. Under 315 nm UV light irradiation, SGV/SV:5% Eu^3+^ shows warm white light emission, consisting of a strong broad band (400–650 nm) with a maximum at 530 nm and some sharp f–f transitions of Eu^3+^ ions with the peaks at 595, 615 and 621, 700 and 705 nm attributed to, ^5^D_0_ → ^7^F_1_, ^5^D_0_ → ^7^F_2_ and ^5^D_0_ → ^7^F_4_, respectively. The f–f transition peak position of Eu^3+^ in SGV/SV:5% Eu^3+^ are similar to those in SGV:5% Eu^3+^, which demonstrates that the Eu^3+^ ions enter the crystal of SGV. However, the relative strengths at 595, 621 and 700 nm of SGV/SV:5% Eu^3+^ are changed, which can be expressed using *I*(^5^D_0_ → ^7^F_1_) : *I*(^5^D_0_ → ^7^F_2_) : *I*(^5^D_0_ → ^7^F_4_), that is, 2.1 : 6.57 : 1. Moreover, comparing with the f–f transition of Eu^3+^ in pure SGV (Fig. 4(b)), whose relative value is 1.74 : 4.48 : 1, the red light was demonstrated to be enhanced in SGV/SV:5% Eu^3+^ compared with the pure SGV:5% Eu^3+^.

The excitation spectrum (Fig. 6(b), blue line) recorded by monitoring the emission of 621 nm (the strongest emission line in the PL spectrum shown in Fig. 6(e)) contains two visible broad absorption bands at 315 and 350 nm as well as some dominated sharp lines in the wavelength region of 350 to 500 nm due to the characteristic f–f transition of Eu^3+^ at about 398 and 469 nm. Under 350 nm UV radiation excitation, the SGV/SV:Eu exhibits green emission, and the obtained emission spectrum consists of a broad band with the peak at 530 nm due to the CT transition of O^2−^ → V^5+^ of SV and very weak f–f transition lines at 614 nm within the Eu^3+^ electron configuration. Thus, SV in SGV/SV can retain its excitation energy for strong green emission and its energy transfer efficiency to Eu^3+^ is lower. Monitored at 530 nm, the excitation spectrum of SGV/SV:Eu^3+^ sample displays a broad absorption band with the peak at 350 nm, as shown in Fig. 6(c), which is similar to the peak of pure SV. This asserts that the broad excitation band in SGV/SV:Eu^3+^ originates from the CT transition of O^2−^ → V^5+^ of SV. Another broad band at 315 nm in SGV/SV:Eu^3+^ must arise from the CT transition of O^2−^ → V^5+^, which has larger blue-shift compared with the O^2−^ → V^5+^ CT transition of pure SGV at 327 nm (Fig. 4(a)). This is due to the change of crystal environment around V atoms and the existence of oxygen deficiency.^29^ The SGV:Eu^3+^ was obtained at 950 °C, and the SGV/SV:Eu was obtained at 750 °C. With increasing calcination temperature, the crystallization of SGV increases, which changes the lattice constant and the oxygen deficiency around V atoms. The change of the lattice constant is small, which generally makes a 1–5 nm shift in CT band, while 5–15 nm blue-shift primarily arises due to of oxygen deficiency.^29^

#### 3.2.4. The dependence of photoluminescence of SGV/SV:Eu^3+^ on the concentration of Eu^3+^

**Fig. 7:**
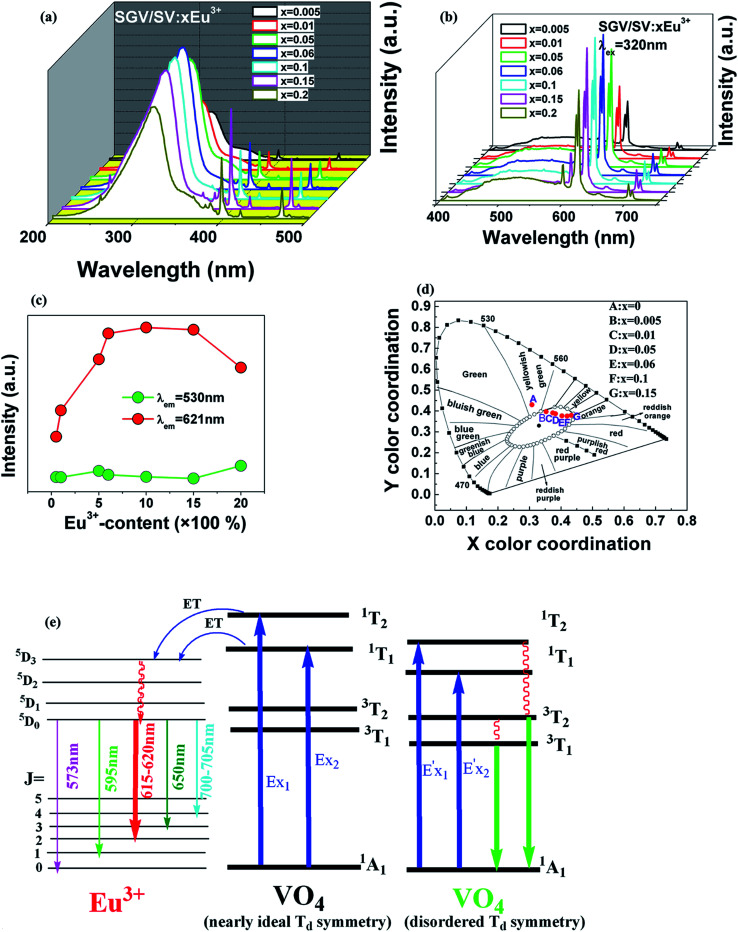
(a) and (b) are PLE (*λ*_em_ = 621) and PL of SGV/SV:Eu^3+^, respectively. (c) The PL intensity of the O^2−^ → V^5+^ charge transfer emission and Eu^3+^-emission. (d) CIE chromaticity diagram showing color coordinates of the luminescence of SGV/SV:Eu^3+^. (e) Schematic model of excitation, energy transfer and emission processes of SGV/SV:Eu^3+^.

Changing the concentration of activator doped in host lattice is a feasible route to realize color-tunable emission; a white emission can be obtained through mixing the green and red light sources at a suitable ratio.^30–32^ In our case, the effects of concentration of Eu^3+^ on the PL excitation and emission spectra of SGV/SV:Eu^3+^ were investigated. Fig. 7(a) and (b) show the variation of PL spectra and emission intensity of Eu^3+^ in SGV/SV:Eu^3+^ samples with the increase of Eu^3+^-doping concentrations from 0 to 20 mol% (*λ*_ex_ = 320 nm), respectively. The changing curve of relative intensity of f–f transition emission of Eu^3+^ at 621 nm (labelled using red circles) and the V–O CT green emission at 530 nm (labelled using green circles) followed the change in concentration of Eu^3+^ as shown in Fig. 7(c). Although the concentration of Eu^3+^ is changed, the emission intensity of V–O CT changed slightly, which indicates that the intensity of V–O CT emission originating from SV in SGV/SV is independent of the concentration of Eu^3+^. This further confirmed that the Eu ions enter the sites of SGV in SGV/SV, while no (or a little) Eu ions can be doped into the sites of SV in SGV/SV. This is because the radius and properties of Eu^3+^ ions are similar to those of Gd^3+^ in SGV; thus, they will occupy the sites of Gd^3+^ ions on priority. The experimental analysis is consistent with the theoretical result. The excitation spectrum monitoring at Eu^3+ 5^D_0_ → ^7^F_2_ at 621 nm clearly shows two broad bands: 200–345 nm (centered at 315 nm) and 345–400 nm (centred at 350 nm), which occur due to the O^2−^ → V^5+^ CT transition of SGV and SV in SGV/SV, respectively. Furthermore, the excitation intensity at 320 nm is stronger than that at 350 nm, which demonstrates that the O^2−^ → V^5+^ CT transition energy can be efficiently transferred to the Eu^3+^ ion. The energy transfer and luminescence mechanism of SGV/SV:Eu is shown in Fig. 7(e). Moreover, the emission intensity of Eu^3+^ first increases with increasing Eu^3+^ from 0 to 15 mol%, and then decreases at 20 mol%. Thus, the emitting color of SGV/SV:Eu^3+^ samples can be tuned through changing the concentration of Eu^3+^. The results can also be confirmed by their CIE chromaticity coordinates shown in Fig. 7(d). The CIE chromaticity coordinates (*x*, *y*) for SGV/SV:*x*Eu^3+^ phosphors excited at 320 nm are listed in Table 4.

**Table 4:** A comparison of the CIE chromaticity coordinates (*x*, *y*) for SGV/SV:*x*Eu^3+^ phosphors excited at 320 nm

Sample no	Sample composition (*x*)	CIE coordinates (*x*, *y*)
A	0	(0.303, 0.436)
B	0.005	(0.349, 0.401)
C	0.01	(0.370, 0.395)
D	0.05	(0.376, 0.391)
E	0.06	(0.406, 0.384)
F	0.1	(0.418, 0.379)
G	0.15	(0.431, 0.382)

Except that the emission of “pure” SGV/SV is at green region, all other SGV/SV:*x*Eu^3+^ phosphors (*x* = 0.005, 0.01, 0.05, 0.1, and 0.15) exhibit warm-white-light emissions. Therefore, we can realize white light in a single component. This component consists of two types of host lattices and one type of rare earth ion activator (Eu^3+^), which can achieve the perfect union between the red f–f transition emission of Eu^3+^ and the O^2−^ → V^5+^ CT transition emission from the two different vanadate host lattices with different [VO_4_]^3−^ symmetries under the UV light excitation. This is a very promising novel method with a wide range of adaptability to obtain white or other color lights.

## 4. Conclusions

1. The vanadate-based phosphors Sr_2_V_2_O_7_:Eu^3+^ (SV:Eu^3+^), and Sr_9_Gd(VO_4_)_7_:Eu^3+^ (SGV:Eu^3+^) and their products Sr_9_Gd(VO_4_)_7_/Sr_2_V_2_O_7_:*x*Eu^3+^ (SGV/SV:*x*Eu^3+^) were prepared by solid-state reaction at low temperature.

2. The bond-energy method is used to investigate the site occupancy preference of Eu^3+^ based on the bond valence model. By comparing the change in bond energy when the Eu^3+^ ions are incorporated into different Sr or V or Gd sites, we observed that Eu^3+^ in SV, SGV or SV/SGV would preferentially occupy the smaller energy variation sites Sr4, Gd and Gd sites, respectively.

3. PL properties of Sr_2_V_2_O_7_:Eu^3+^ (SV:Eu^3+^) and Sr_9_Gd(VO_4_)_7_:Eu^3+^ (SGV:Eu^3+^) and their products Sr_9_Gd(VO_4_)_7_/Sr_2_V_2_O_7_:*x*Eu^3+^ (SGV/SV:*x*Eu^3+^) were investigated, which shows their excitation and emission characteristics. Their excitation wavelengths ranging from 220 to 400 nm fit well with the characteristic emission of UV light-emitting diode (LED) chips. Two broad charge transfer bands arising from O^2−^ → V^5+^ of [VO_4_]^3−^ with the peaks at about 325 and 350 nm coexist in SGV/SV:Eu^3+^, which indicates that the phosphors can be efficiently excited in the UV region.

4. Eu^3+^ ions primarily enter the sites of Gd^3+^ of SGV. The photoluminescence properties of SGV/SV:Eu^3+^ component shows two prominent properties: first is that V–O CT energy of SGV can be efficiently transferred to Eu^3+^ and strong red photoluminescence can occur coming from the ^5^D_0_ → ^7^F_2_ transition of Eu^3+^; second is that the intensity of V–O CT transition emission originating from SV is not influenced by the concentration of Eu^3+^ and retains green light emission.

5. With the increase in concentration of Eu^3+^ from 0.5 to 15 mol%, the intensity of red f–f transition of Eu^3+^ at 621 nm increases, and concentration quenching was observed 20 mol%. All the SGV/SV:*x*Eu^3+^ (*x* = 0.005, 0.01, 0.05, 0.1, and 0.15) phosphors exhibit warm-white-light emission.

## Conflicts of interest

There are no conflicts to declare.

## Supplementary Material

RA-008-C7RA08089A-s001
