# Supplementary material for: Preferential occupancy of Eu3+ and energy transfer in Eu3+ doped Sr2V2O7, Sr9Gd(VO4)7 and Sr2V2O7/Sr9Gd(VO4)7 phosphors
Source: RSC Adv. 2018 Jan 3;8(3):1191–202. doi: 10.1039/c7ra08089a (PMC9077092; doi:10.1039/c7ra08089a)
Supplement: RA-008-C7RA08089A-s001 [file RA-008-C7RA08089A-s001.pdf]

Electronic Supplementary Material (ESI) for RSC Advances.
This journal is © The Royal Society of Chemistry 2017

Supplementary materials

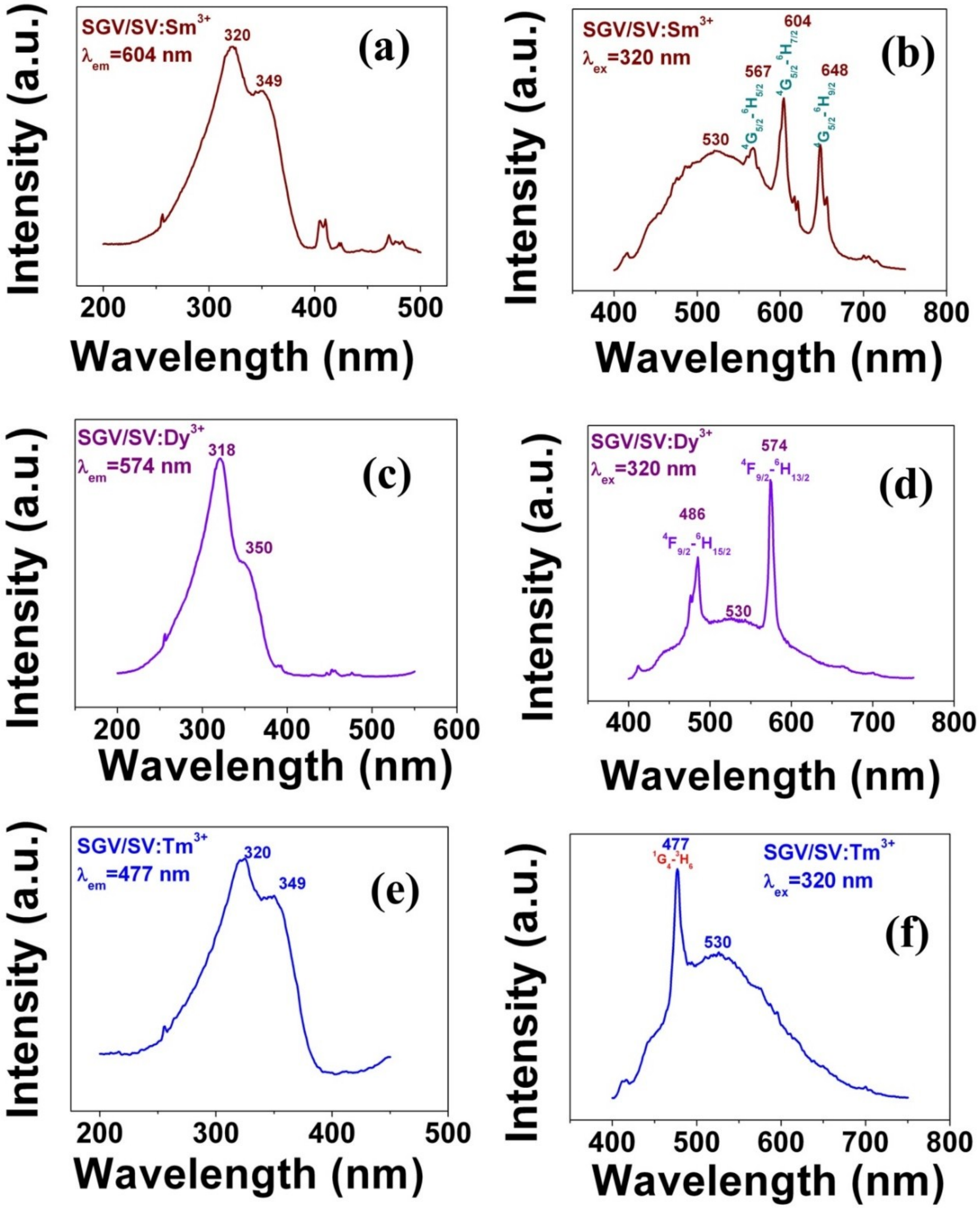

Figure S1. PL excitation and emission spectra of SGV/SV:Ln^{3+}.(a) and (b): SGV/SV:Sm^{3+}; (c) and (d): SGV/SV:Dy^{3+}; (e) and (f):SGV/SV:Tm^{3+}.
